# Strategies for Creating Robust Patient Groups to Study Diverse Conditions with Electronic Health Records

**DOI:** 10.1146/annurev-biodatasci-020722-114525

**Published:** 2025-04-08

**Authors:** Grace D. Ramey, Hannah Takasuka, John A. Capra

**Affiliations:** 1 Biological and Medical Informatics Program, University of California San Francisco, San Francisco, California, USA; 2 Bakar Computational Health Sciences Institute, University of California San Francisco, San Francisco, California, USA; 3 Oral and Craniofacial Sciences Program, University of California San Francisco, San Francisco, California, USA; 4 Department of Epidemiology and Biostatistics, University of California San Francisco, San Francisco, California, USA

**Keywords:** electronic health record, case-control studies, cohort studies, bias epidemiology, feasibility studies, reproducibility of results

## Abstract

The growth of electronic health record (EHR) databases in size and availability has created an unprecedented opportunity to better understand human health and disease. However, conducting robust EHR studies requires careful filtering criteria and study design, as EHRs pose several challenges that can confound analyses and lead to inaccurate results. Here we review these challenges and make suggestions about how to avoid or adjust for major confounders and biases in common EHR study designs. We further highlight qualities of EHR data that make different diseases more or less feasible for study. These recommendations for conducting research using EHRs will help inform database selection, improve reproducibility of results across the field, and enhance the validity of study results.

## 1. INTRODUCTION

Electronic health records (EHRs) are a rich data resource for biomedical studies and have numerous advantages for research. They are cost-effective compared to prospective clinical studies, where patient recruitment, outlining protocols, and supplying treatments each require substantial financial resources. They also consist of real-world data and can be used for retrospective analyses, allowing scientists flexibility to conduct studies ranging from simulated randomized drug trials to disease biomarker discovery and risk factor analyses. EHRs are increasingly available through growing clinical biobanks and institutional health centers, making deidentified patient data accessible to researchers from around the world. Further highlighting the growth in EHR use for research, a recent PubMed search for the phrase “electronic health record” indicated that the number of EHR and related publications has increased substantially in the past decade, from fewer than 6,000 EHR studies published in 2014 to over 10,000 published in 2024.

The numerous advantages of EHR research and advances in epidemiological biostatistics (1) have promoted the use of EHRs, but limitations remain. Several types of bias are pervasive in the construction of patient study groups from EHRs. These problems can cause errors in statistical analyses and decrease overall accuracy and reproducibility of research results, ultimately invalidating studies’ conclusions. Additionally, certain types of conditions are more likely to be misreported or remain uncaptured due to both the structure of EHRs and larger healthcare practices. In this article, we discuss these and other challenges that must be accounted for in EHR studies. We review the ways in which these limitations present themselves in the literature today, and we discuss how researchers have addressed them. We also make recommendations for approaches to reduce biases, inaccuracies, and confounders in EHR study designs and to ensure robust results through multiple validation strategies. Furthermore, we highlight factors that make some conditions more conducive (and others less so) to study with EHRs to give researchers a broad sense of the feasibility of different EHR studies. With these pitfalls, strategies, and background knowledge in mind, we emphasize the importance of conducting sound EHR studies for the biomedical research community.

## 2. A BRIEF OVERVIEW OF COMMON EHR STUDY DESIGNS

One of the first uses of EHRs in biomedical research occurred in the 1960s, when researchers determined the likelihood of bronchial asthma in patients given questions about respiratory symptomatology (2). Patients answered questions using punch cards that could be read into a computer and later used for inference. With the US government’s push to improve healthcare quality using electronic systems starting in the 2000s (3), EHRs have become vastly more advanced and ubiquitous. Virtually all categories of healthcare providers have increased their use of EHRs over the last decade, including providers in public hospitals, private office settings, and even long-term residential care facilities (4–6). With EHR use becoming widespread, the range of conditions that can be studied has expanded dramatically; research now commonly addresses the diversity of human conditions from cancer (7) to COVID-19 (8) to neurodegenerative diseases (9). As EHRs constitute real-world data that have not been curated for a single study, a range of observational study types can be conducted using these large clinical databases, often providing complementary ways to study ailments from multiple perspectives.

We briefly cover observational study designs commonly constructed in EHR research and the corresponding types of questions best suited to them. We devote a particular focus to study designs that are commonly used for risk analyses in biomedical informatics. For brevity, we refer to the structures of patient groups that one can curate from EHR databases as study designs, while study types refer to the follow-up analytical approaches that investigators can apply to the resulting patient groups.

We cover three main observational study designs in EHRs: case-control, cohort, and cross-sectional studies (Figure 1). In case-control study designs, researchers select patients from the EHR based on their outcome status, e.g., the occurrence of a particular disease, followed by the determination of whether patients had a particular exposure of interest. For example, Ganz et al. (10) conducted a case-control study by selecting patients based on their type 2 diabetes status and validated the association between high body mass index and increased risk for type 2 diabetes as one of the many investigations highlighting the relationship between metabolic phenotypes and diabetes risk. Because the outcome is the defining inclusion feature in case-control studies, these observational designs can be especially useful when studying rare diseases that may appear only in small numbers in the general population.

In contrast, in cohort studies, patients are selected based on whether they had an exposure of interest, such as a drug treatment or the presence of a potential risk factor, like a hazardous environment. Patients with the exposure are assessed after inclusion for occurrence of a later outcome, such as a particular disease or lab measurement. For example, Brooks et al. (11) used this design to highlight the relationship between atrial fibrillation (AF) and dementia incidence after selection of patients based on their atrial fibrillation status, and they found greater dementia incidence in those with AF. Case-control and cohort studies are both commonly used in EHR research because they allow scientists to study exposure–outcome relationships over time and therefore establish putatively causal links between two clinical measures. We focus primarily on these study designs throughout the remainder of the review, but briefly, cross-sectional studies are a third type of observational study design that involve the analysis of exposures and outcomes at a single point in time. There are no inclusion criteria for patients based on exposure or outcome, and therefore these studies are often used to determine general prevalence statistics. As a result, determining causal relationships with cross-sectional studies is challenging (12).

After a group of patients has been identified using one of these observational designs, researchers can make use of several different study types that suit many styles of research questions. Risk analyses are very common in EHR research. In risk studies, researchers compare the risk of developing an outcome in an exposed group versus an unexposed group. These studies often mimic randomized controlled trials; hence, the two exposure comparison groups may often be referred to as treated and nontreated groups. Multiple statistical measures of risk can be used in these study types, including odds ratios, relative risk, and hazard ratios, and the correct choice of statistical measure will depend on the study design. We refer readers to George et al.’s (13) work on the relative merits of each of these measures and the study designs they can be used for. Recently, researchers have expanded risk analyses across traits and discovered associations between immune-related exposures like viral infection or autoimmunity and neurodegenerative disease occurrence (14–16). By extension, these and related cross-trait studies may suggest potential treatments for risk-elevating traits as interventions for downstream outcomes. Risk factor studies can also be used in a more targeted fashion, for example, for drug repurposing to test if specific medications are associated with decreased risk of an outcome like surgery, relapse, tumor growth, or disease development (17–19). For conditions that are particularly difficult to treat, this type of study can provide biological insight and potential benefit to patients. For example, the causes of Alzheimer’s disease are not fully understood and there is no single clear treatment path for patients. As a result, it has been the focus of several risk and drug repurposing studies. In a risk study involving a cohort meta-analysis, Zhang et al. (20) demonstrated that the use of nonsteroidal anti-inflammatory drugs was linked to a nearly 20% reduced risk of Alzheimer’s disease. In a multisite drug repurposing study, bumetanide (21), a medication typically used to treat hypertension, was also associated with decreased risk of Alzheimer’s disease. Other observational risk and drug repurposing analyses in clinical databases have focused on Parkinson’s disease (22), Crohn’s disease (19), and more, highlighting the great benefit that these study types can provide.

In addition to risk analyses, several other study types are common in EHR data. These involve methods to predict disease onset based on biomarkers using techniques like machine learning (23), identifying heterogeneous disease phenotypes using latent factor decomposition (24, 25), or advising patients on medical conditions using natural language processing (NLP) and generative artificial intelligence (26). While we do not focus on these topics here, they further represent the breadth of possibility when it comes to EHR study types.

Risk analyses have led to substantial insight from EHRs, but there are unique properties of EHRs and study cohort creation steps that can affect these types of analyses. In the following section, we explore and categorize these challenges and propose strategies for addressing them.

## 3. CURRENT CHALLENGES IN STUDY COHORT CREATION

### 3.1. Selection Bias

Observational studies inherently require the selection of participants from an existing pool of patients, and therefore they are susceptible to selection bias. In the following sections, we discuss challenges and current solutions to the selection bias problem.

#### 3.1.1. Challenges.

Selection bias occurs when patients selected for a study are not representative of the underlying target population they were sampled from. This can occur when the exposure and outcome are linked in such a way that makes patients more likely to be selected for one group when they are part of the other. Often in cohort studies, patients may be more likely to be selected for the exposure group if they have the outcome, and in case-control studies, patients may be more likely to be selected for the outcome group if they have the exposure. This can produce baseline differences between groups that can inflate calculated risk among patients, and there have been studies highlighting many factors that can influence selection in widely used clinical databases, such as the UK Biobank (27). Hernán et al. (28) described in detail an example case-control EHR study of heart attack risk where an extraneous condition that is related to both the outcome and the exposure can cause selection bias. In this study, the outcome of interest is a heart attack, and the exposure of interest is the use of postmenopausal estrogens. In this example, researchers selected their controls (patients without heart attacks) from a group of people that happened to have a higher incidence of hip fractures (the extraneous condition) than the cases (patients with heart attacks) due to disparities in the clinic data covered by the EHR. Because taking estrogens lowers the incidence of hip fractures (29, 30), patients in the control group were less likely to have taken estrogens. This creates a situation in which the selected controls are less likely to have had the exposure, making it appear as though estrogens are associated with heart attack when they actually are not. Similar bias effects of extraneous conditions can also occur in other observational study types (31, 32).

Survivorship bias is another type of selection bias that often appears in retrospective observational studies. Survivorship bias occurs when a patient’s record is missing data for a nonrandom reason, and that patient passes selection criteria because of the missing data. For many studies, this arises when investigating conditions that significantly alter the lifespan of patients (33, 34). Consider a retrospective cohort study of patients with autoimmune diseases constituting the exposure group. Autoimmune diseases can lead to increased mortality and shorter lifespans in those that are affected (35, 36), and this could result in more exposed patients passing away (or dropping out) of the study prior to having a chance to develop a later outcome (e.g., a late-onset condition like Alzheimer’s disease). In a risk analysis, this phenomenon could skew study results to appear as though exposed patients are less likely to develop Alzheimer’s, when, indeed, the opposite may be true. Survivorship bias and missing follow-up for other nonrandom reasons are important challenges to account for when constructing study groups.

#### 3.1.2. Solutions.

Directed acyclic graphs (DAGs) are useful tools for mapping relationships between variables that may influence both exposures and outcomes in observational studies (28, 37, 38). Variables that often link the exposure and outcome in a noncausal way include sociodemographic factors as well as other phenotypes that may act as known or unknown comorbidities. Similar to hip fractures in the aforementioned example, Rusconi et al. (39) made use of DAGs to highlight an association between antibiotic use and asthmatic symptoms in childhood that was confounded by infections as an extraneous variable (Figure 2). DAGs can also be useful for highlighting potential missing data in individuals that could result in survivorship bias and loss to follow-up cases.

In addition to visually mapping out relationships, researchers may also be able to quantify the amount of selection bias present in their study, depending on the nature of their data. Criqui (40) and Austin et al. (41) pioneered the use of the relative odds ratio in the late 1970s and early 1980s as a way to determine how much of the computed risk in an observational study is due to selection bias and how much is due to a true observed effect size. Relative odds ratios are computed by comparing the odds ratio (i.e., the risk of an outcome) in the selected population to the odds ratio in the background, or nonselected, population. A relative odds ratio of 1 indicates that no selection bias occurred, while a relative odds ratio greater or less than 1 indicates that the true effect size was over- or underestimated, respectively (37). Nohr & Liew (37) further highlighted that the same logic can be used for estimating the prevalence of conditions in a selected population compared to the prevalence in the population as a whole.

While the relative odds ratio quantifies selection bias, it assumes that the data of individuals in the nonselected population are not subject to other biases, such as differences in healthcare utilization, which, as we discuss in later sections, are common in EHR data. Furthermore, in retrospective EHR studies where the data researchers work with are often deidentified and cannot be linked back to individual patients, it can be infeasible to obtain follow-up information from the nonselected population to determine an accurate representation of risk or prevalence. Given these challenges in estimating selection bias in EHRs, we recommend that EHR researchers should always adjust for selection bias using the techniques discussed below.

In statistical epidemiology, data matching techniques are used to ensure that exposed and unexposed groups do not differ significantly from each other in terms of their background covariates. In other words, the exposed and unexposed individuals should only differ from each other randomly (42), as any systematic differences between the groups could alter effect size estimates. In matching, differences, or distances, between exposed and unexposed individuals are calculated based on covariates selected by the researcher, and these distances can then be used to match exposed individuals to unexposed individuals (42).

Propensity score matching is a common and powerful statistical approach for creating matched cohorts that involves calculating the probability that an individual has the exposure of interest based on a logistic regression of their covariates (43) (Figure 3). Exposed and unexposed individuals are then matched based on propensity score similarity. Another common type of matching, Mahalanobis matching, directly determines the distance between sets of covariate values, and, unlike propensity score matching, patients are matched on these covariate distances rather than on an aggregate score (44) (Figure 3). Mahalanobis matching can further be used to characterize the distance between a patient’s covariates and a larger underlying distribution of covariates, and matching can be performed based on this distance or the distance between the covariate vectors of two individual patients. Finally, exact matching entails a direct 1:1 agreement of covariates between an exposed and unexposed individual in order for them to be matched (Figure 3).

Each of these matching methods has advantages (45–47) and disadvantages (48, 49), and methods have even been combined in past studies to try to obtain the best matches possible (16, 50, 51). Exact matching is ideal in cases where specific binary covariates, such as sex (16), need to be exactly the same among exposed and unexposed individuals because they will be investigated in downstream analyses, as opposed to covariates that may be allowed to demonstrate more variation, such as birth year or lab measurements. Mahalanobis matching too is useful because it involves the minimization of the distance between raw covariates, often resulting in a good balance between matched pairs. Both methods can additionally be used on categorical and continuous covariates. However, both exact matching and Mahalanobis matching run into issues when the number of covariates becomes large. An increasingly long list of covariates decreases the likelihood that an exact match will be found for every variable between patients in the case of exact matching, or, in the case of Mahalanobis matching, the computational complexity becomes too great when trying to minimize many distances among raw covariates. Propensity score matching does not suffer from this limitation, as covariate differences between patients are condensed down to a single propensity score value rather than individual covariate distances. While there is debate over the merits of condensing information into a single score (45, 48), this allows the propensity scoring method to have great versatility in handling large patient sample sizes with many covariates, and it is therefore commonly used to combat selection bias in observational EHR research (52, 53). Overviews and pros and cons of each method are highlighted further in Figure 3.

The propensity scoring technique can be further extended with the inverse probability of treatment weighting (IPTW) method. As described by Chesnaye et al. (54), Joffe et al. (55), and others (56), in this method, patients are weighted by the inverse of their propensity scores, such that exposed patients with a low probability of exposure based on their covariates are upweighted, and unexposed patients with a high probability of exposure are upweighted. The resulting weights are applied to patients, creating what is often called a pseudopopulation of people whose covariates are balanced across exposures and whose weights suggest they contribute more or less to the final effect size estimate than a normal patient would. Typically, a weighted linear or logistic regression model is then applied to the pseudopopulation, which results in odds ratio or other risk effect estimates (57, 58). It is important to note that IPTW can suffer from extreme weight values if covariates are very different between populations (59), leading to extremely high or low propensity scores that increase exponentially when inverted. To avoid this, using standard propensity scoring or stabilizing or truncating IPTW weights are useful options for further balancing groups of patients before conducting inference (59, 60).

Accounting for selection bias in EHR studies is essential to improve the clinical applicability of discoveries. External factors like sociodemographic variables, other phenotypes, and differential survivorship or follow-up can all influence exposure–outcome relationships and therefore the selection of patients for a study. Teasing these variables apart, while potentially statistically complex and computationally intensive, is necessary to identify reproducible and accurate associations within clinical data. We and others (42) stress the need to test for a balance of covariates between exposed and unexposed groups by looking at measures of distance between matched pairs, and we suggest that adjustments are made accordingly using the aforementioned techniques. In later sections, we also discuss how data matching techniques are useful for addressing other types of bias.

### 3.2. Information Bias

In addition to selection bias, information bias is also common in observational EHR studies. Below we discuss challenges stemming from this source of bias and ways to mitigate the confounding that it may cause.

#### 3.2.1. Challenges.

Information bias results from any EHR inaccuracy caused by data missingness or data entry errors that can propagate into downstream analyses (61). Phenotyping errors, also termed misclassification bias, are one source of information bias, and they are a major reason for lack of replication across EHR studies (61). Errors in determining whether a patient exhibits a particular phenotype can stem from differences in diagnosis criteria for various conditions or from differences in specificity of the criteria. O’Malley et al. (62) highlighted the example of cancer versus schizophrenia. Given that lab measures related to cancer tissue histopathology are more concrete with accepted standards for diagnosis compared to the more variable behavioral and psychological tests that can lead to a schizophrenia diagnosis, cancer tends to be diagnosed with greater accuracy than schizophrenia. Phenotyping errors can also stem from provider preferences and specificity when it comes to selecting relevant medical billing codes (63). A provider at one hospital may select a very specific billing code if a woman pregnant with twins were to be treated, whereas another provider may not. The International Classification of Diseases (ICD) code ICD10 O30.031, corresponding to a twin pregnancy in the first trimester, encodes much more information than a code like ICD10 O30.90, corresponding to an unspecified multiple gestation. The difference in information contained within each of these codes can lead to vastly different numbers of patients being included in research studies, which may affect the distribution of the overall study population. Providers may also select billing codes that are more likely to be reimbursed or that are only relevant to the patient’s immediate ailment, which can lead to systemic insurance and medical code selection biases that may affect research studies (61).

Information bias can also result from data missingness. Fragmentation is the missingness that occurs when a patient is seen or treated at one healthcare system but switches healthcare systems at a later time point, often resulting in a lack of continuity of care (Figure 4a). Due to the absence of data integration across many healthcare systems, especially in the United States (64–66), this makes it difficult for researchers to gain an accurate representation of a patient’s full longitudinal health trajectory. This was apparent in a clinical phenotyping study by Wei et al. (67), where as many as 33% of patients were incorrectly phenotyped for type 2 diabetes when they were seen at more than one medical center. Apart from diagnoses being wholly missed due to data fragmentation, diagnoses that are captured across healthcare systems may also exhibit inaccuracies in their diagnosis dates. Consider a patient with a chronic condition that persists for more than 10 years (Figure 4a). If the patient were to enter a new healthcare system for the first time 5 years after their condition first appeared, the diagnosis date of the condition could be logged as 5 years after the actual start date in that new healthcare system. If the patient were to then enter a different healthcare system 5 years later, the condition could again be logged with a diagnosis date much later than the actual start date, now with a delay of 10 years at the second healthcare system. For researchers who rely upon the timing of diagnoses as factors in their analyses, this type of fragmentation can pose major challenges, as different healthcare system databases can represent very different pictures of patients’ true health trajectories (Figure 4b).

If patients were to stay at a single healthcare system throughout their lifespans, data missingness could also arise due to differences in healthcare utilization among people. The frequency of data capture would be the same if all patients visited the doctor roughly the same amount, but many factors influence how much patients engage with healthcare systems, and it is important to be aware of these when constructing cohorts. Disease severity can influence the amount of information available on each patient. Studies of several conditions including systemic lupus erythematosus (68) and nonalcoholic steatohepatitis (69) have indicated that people with more severe forms of diseases have increased numbers of primary, outpatient, and emergency room visits compared to patients with milder forms of disease, potentially leading to more frequent and comprehensive data capture for these individuals. Increased healthcare utilization can be further compounded by the increased presence of comorbid conditions in those that have chronic diseases (70). Notably, there is also evidence that severe disease can influence healthcare utilization patterns in the opposite direction by increasing data fragmentation (71), because patients with more severe forms of certain conditions may be referred more often to external healthcare systems that can provide specialized care. As discussed further in Section 3.3, social determinants of health can also influence healthcare utilization (72) through factors like proximity to a healthcare site, financial capability to pay for services, health literacy, and language compatibility with healthcare providers. For example, Samuelson et al. (73) discovered that individuals living in zip codes consisting of higher education rates and income were more likely to seek rhinology care, while Jones et al. (72) determined a similar phenomenon when studying cumulative social burden due to the need for assistance with food and housing in different patients. These can ultimately lead to differences between patient groups that could affect the validity of the results of a research study. We now discuss several strategies to navigate issues that information bias can present.

#### 3.2.2. Solutions.

In many EHR studies, the effects of information bias on phenotyping accuracy are evaluated by conducting chart reviews (74). In a chart review, a clinician checks the complete longitudinal EHR of a patient (including unstructured clinical notes) to determine if there is evidence for a diagnosis or procedure that matches what was algorithmically assigned (usually based on the patient’s structured billing codes). We recommend that EHR researchers employ the expertise of clinicians throughout all stages of studies, but this is particularly important during study group creation. Expert chart review on a subset of the records of patients with the relevant exposures and outcomes is necessary to ensure that disease classifications are sufficiently accurate for the study. Even if chart review is conducted after study group creation and follow-up analyses, it can quantify the accuracy of the starting population and therefore aid interpretation of the study results. Castro et al. (75) described the use of chart review for identifying the validity of bipolar disorder diagnoses. A subset of roughly 200 individuals out of a total study group population of ~50,000 was selected for chart review, and phenotype misclassification was quantified using the positive predictive value (PPV) (76), or the fraction of patients correctly phenotyped with bipolar disorder out of the total number of patients phenotyped with the disorder. Reported PPVs from studies in the literature can vary, ranging in some cases from 40%, for example, in a study of autism by Perochon et al. (77), to over 70% in others (78), and even over 90% (79, 80) still in others. While there is no clear threshold on PPV to indicate success, ultimately, the higher the PPV the better. PPV can also vary depending upon the nature of phenotyping algorithm used or the condition being studied. In the aforementioned example, while a condition like cancer may be easier to concretely diagnose, conditions like schizophrenia may present significantly more variability in terms of the billing codes and clinical notes used to definitively identify them, and it is necessary to keep this condition-specific misclassification in mind when interpreting PPV and other phenotyping statistics. Other statistics such as sensitivity (true positive rate) are also important to consider, as these are unaffected by the overall prevalence of a condition or procedure, which PPV can be susceptible to (74). Ultimately, accuracy estimates from chart review that evaluate the trade-off between sensitivity and specificity provide necessary context for statistical power calculations and study interpretation.

Manually evaluating hundreds or thousands of patient records to determine accuracy of diagnoses and procedures is extremely labor- and time-intensive for clinicians (81). As a result, manual chart reviews typically only account for a small fraction of the records relative to the total sample size. Recently, researchers have turned to NLP algorithms to simulate manual chart review and increase the accuracy and sample size when phenotyping. Wi et al. (82) demonstrated that NLP-based phenotyping algorithms can obtain agreement as high as 96% with manual chart review results, while Khurshid et al. (83) highlighted that NLP algorithms can add to study sample sizes by as much as 25% through the identification of cases that would otherwise have been missed. As NLP algorithms continue to progress, their application to improving EHR phenotyping will be imperative, and there are many opportunities for their integration with manual chart review in current and future studies.

There are several clinical databases, such as the All of Us cohort and the UK Biobank, that do not provide access to clinical note texts, only structured billing codes, which makes it difficult to apply NLP or conduct chart reviews in these situations. In these cases, there are other ways to mitigate information bias stemming from inaccurate phenotyping including validating combinations of codes that occur in patients’ records that, in aggregate, are likely to point to a particular diagnosis or condition. Weinmann et al. (79) highlighted that using two or more billing codes related to diagnoses and procedures dramatically increased the PPV of a study identifying pediatric leukemia patients. Bastarache (84) further described the usefulness of manually aggregated schemas such as phecodes that provide groupings of ICD codes that point to clinically meaningful concepts and diagnoses. These aggregated phecodes can help translate the relatively noisy landscape of EHR billing codes into more relevant phenotypes for biomedical researchers.

Information bias resulting from care fragmentation can be measured using indices such as the Bice–Boxerman Continuity of Care Index (85), the Sequential Continuity Index (86), and the Usual Provider of Care Index (87), among others (88, 89). Many of these indices rely on knowledge of patients’ healthcare utilization in other healthcare systems. Thus, it can be difficult to measure these in an EHR study at a single healthcare system, which is commonly all that is available for many EHR researchers. As such, we reiterate our suggestion to use data matching techniques to adjust for fragmentation of care within a single healthcare system and study. Variables that can help account for care fragmentation, because of either a patient moving healthcare systems or differential healthcare utilization, include a patient’s total number of visits in a healthcare system, frequency of visits per year, number of referrals, and length of record (last visit date minus first visit date) (16, 90). Analyzing study groups both with and without these variables present in matching is essential, as they can act as both confounders and colliders. While a confounder is a variable that influences both the exposure and outcome, a collider variable is influenced by the exposure and the outcome (91). In the case of healthcare utilization at a single hospital, consider matching exposed and unexposed patients based on the frequency of visits. This would in theory capture exposed and unexposed patients who sought care the same amount, thereby ensuring that patients in the study had equal chances of being diagnosed with a later outcome and removing healthcare utilization as a confounder. However, by enforcing that the unexposed patients have the same visit frequency as those who are exposed, we may also be increasing the inclusion of patients who have other comorbidities that cause them to seek care more often in the unexposed group, thereby representing a relatively unhealthy matched group. In this case, healthcare utilization could be acting as a collider. Because of this dichotomy, the strongest study results are those that remain similar when accounting for care fragmentation and healthcare utilization using matching to adjust for both cases, and effect size estimates should also remain similar in both analyses.

### 3.3. Diversity and Access to Care

Lack of diversity and differential access to care can confound EHR study findings. Below we cover ways that these variables may affect study designs and results, as well as ways in which researchers can account for diversity and access to care in their investigations.

#### 3.3.1. Challenges.

The relevance of EHR research findings to the general population is limited by the representation of different groups within EHR data. Many research publications do not address the presence and implications of bias within the individuals in their cohorts. In reality, a majority of clinical databases contain relatively homogenous populations. This propensity of EHRs to lack diversity, in addition to containing racial/ethnic biases, can stem both from the interaction of providers and patients with EHRs and from the inherent structure of EHRs. The interfaces where clinical providers enter information into the EHR about patient race, ethnicity, gender, sexual orientation, diagnoses, and other personal characteristics are created by humans. Thus, the presentation of different categories and information to providers and patients will be inherently shaped by human biases, whether or not the designer is aware of them (62, 92). The very presence of categories for multiple types of demographic information can perpetuate biases as well, as patients may feel as though the existing delineations for demographic fields are not a perfect fit to their identity. Indeed, self-reported demographic information in EHRs based on existing structures is not the same as free responses from patients, and it may obscure greater diversity. Particularly for demographic fields like race or ethnicity, it is important to note that these are also not true estimations of genetic ancestry, and it is crucial to keep this lack of granularity in mind when handling diversity in EHR data.

Lack of diversity in EHR data is also perpetuated by differences in propensity and ability to use healthcare. Social determinants of health (93) such as proximity to a healthcare provider, ability to afford care, and knowledge of health information or disinformation can all affect access to care and therefore the propensity to use it. Many studies (72, 94) have highlighted the worse health outcomes, both mental and physical, that patients with greater social needs experience. Even if healthcare is relatively accessible, patients may be reluctant to engage with the healthcare system because of past negative experiences and mistrust in clinicians, and this can also ostracize particular populations of patients (95). Rozier et al. (92) highlighted the example of improper documentation of a patient’s past medical history, which can lead to providers not having enough information to make medical decisions and ultimately worse care for the patient. This can begin a negative cycle where a patient may feel they are receiving inadequate care, and they may be less likely to visit a clinician in the future, ultimately feeding into the lack of available data. Similarly, if a patient feels they will not be taken seriously or have their symptoms believed, it can sow mistrust in not just a single clinician, but the healthcare system as a whole, perpetuating the lack of inclusion of certain patients in EHR data (96). Marginalized minority populations are the most commonly excluded, and they tend to be undertreated and misdiagnosed at greater rates in comparison to white patients (97, 98). They similarly tend to experience greater data fragmentation compared to other groups (71, 99). This is why it is of the utmost importance for providers and researchers alike to be aware of the ways in which biases and a lack of diversity can influence EHR data, and it is essential to be mindful of these factors when designing and interpreting EHR studies.

#### 3.3.2. Solutions.

Just like selection and information bias, a lack of diversity in EHR data can bias results and limit transferability of conclusions across studies. Therefore, it is imperative that EHR researchers are clear and detailed about the characteristics of their study populations as they make conclusions in their work. To increase diversity from the start, researchers can choose to balance sample sizes across different demographic populations. We also encourage researchers to externally validate their research in other healthcare systems or clinical databases (see Section 5.2). Selecting an external database for validation that contains different population demographics than the original database is one way to determine if results generalize across more than one population. Additionally, while there should be diversity in observational study groups, it is important to account for it in analyses, due to the confounding it can present from differing propensity for healthcare utilization, fragmentation, insurance status, and other social factors among different populations (71, 72, 100), as previously discussed. Including social determinants of health and other demographic categories that are as granular as possible as variables to match or regress on provides insight into how diverse social factors may contribute to the outcome of interest.

As more EHR studies are conducted every year, researchers have an obligation to unambiguously state the demographic characteristics of their study groups. This ultimately helps the field by identifying unanswered research questions that may still exist in understudied groups and previously uncharacterized areas of social need that may influence results. Furthermore, EHR studies have the potential to highlight gaps in the ways that EHR structures document diversity, and they can shed light on the need for necessary changes to these structures that will perhaps make them more patient-centered and well-suited for the full extent of human diversity in healthcare in the future (101).

## 4. FEASIBILITY OF EHR STUDIES VARIES BY DISEASE

EHR studies are more feasible for certain conditions than others, given documentation patterns in the EHR. In this section, we discuss several dimensions along which conditions vary that contribute to EHR data quality and therefore study feasibility. We also stress that, for conditions that pose challenges when being studied using EHR data, there is still great promise and opportunity for methodological innovation and biomedical research, but extra care is warranted to ensure the captured data are as accurate as possible and that all limitations are well-described. We also note that not all of these dimensions that influence feasibility have been comprehensively studied, so we additionally suggest hypotheses and avenues for future work.

### 4.1. Disease Prevalence and Clarity of Disease Definition

Conditions that are less prevalent, such as rare diseases, can prove challenging to work with in observational EHR studies. If a condition is exceedingly rare, there may not be a large enough sample size to conduct a well-powered study, even in large EHR databases with millions of patients. For this reason, it is important to conduct statistical power calculations early on in a study to ensure appropriate power and high confidence in results. Patients with rare conditions may also have a delay in diagnosis or many misdiagnoses leading up to a final accurate diagnosis (102, 103), further muddling the true phenotype and EHR trajectory. In recent years, EHR researchers have leveraged machine learning to more accurately diagnose rare diseases. Lo Barco et al. (104) used NLP to extract structured phenotype information from clinical notes on the rare disease Dravet syndrome, which typically exhibits a delay in diagnosis of 2 years on average. Using machine learning, they identified clinical concept language that occurred more often in cases of Dravet syndrome, and knowledge of these concepts could aid in earlier and more accurate diagnosis of the disease (105, 106). Analyzing the rich phenotypic information present in EHRs can also help identify individuals with constellations of rare phenotypes who would benefit from more rigorous diagnostics such as genetic testing (107). These examples illustrate how using powerful phenotyping algorithms can aid in the use of EHRs for rare disease research in the future. On top of this, different bodily systems and conditions can have differential coverage in the EHR compared to their prevalence. These kinds of algorithms and phenotyping strategies may be useful in bypassing these data challenges.

Even for common conditions, difficulties can arise in EHR studies if the condition is poorly defined or shares symptoms with many other diseases. Mental health conditions are a prime example, as their diagnosis can entail variable testing strategies and different clinical thresholds for diagnosis from hospital to hospital (108). Autoimmune diseases pose similar challenges due to the fact that many symptoms overlap between them, such as skin sensitivity, joint pain, and fatigue (109). Patients with autoimmune diseases exhibit on average a 4.5-year delay in diagnosis time (110), in part due to this symptom overlap. Delays in diagnosis can also make a patient’s health record susceptible to error because of improper treatment courses and potential opportunity for care fragmentation.

Given these challenges that disease prevalence and clarity of definition can pose for EHR studies, how can researchers ensure their work is robust and accurate? For particularly rare diseases, incorporating data from a disease-specific database rather than or in addition to a general EHR database can help ensure accurate phenotyping and appropriate controls. Rare disease databases that may be leveraged include the Undiagnosed Diseases Network database (111), the Online Mendelian Inheritance in Man database (112), and databases consolidated by the Orphanet consortium (113), among others (114–116). To address the presence of misdiagnosis and delays in diagnosis, chart reviews to verify accuracy in diagnosis and diagnosis timing are essential. We also anticipate that advanced algorithmic approaches from NLP and artificial intelligence will enable extraction of structured information and interpretation of rare disease diagnoses from clinical notes more regularly in the coming years. Despite rare and ill-defined conditions being challenging, as long as researchers clearly convey the limitations, these studies are vital to advancing our understanding of the biology of disease and patient care.

### 4.2. Data Richness and Care Setting

Frequency of doctor visits also influences documentation quality in the EHR. Conditions that require more frequent interactions potentially lead to a more data-rich EHR due to the greater number of clinical measurements and check-ins across the lifespan. Consistent quantitative clinical data like blood lab values that are an example of continuous measures of a patient’s health status are valuable windows into the temporal variation in many conditions. Greater data richness across time may also increase the likelihood that a disease is recognized by a clinician or captured accurately, as clinicians will have more interactions to recognize abnormalities in a patient’s health state and have access to a greater amount of data to make informed decisions about diagnoses. This greater phenotyping accuracy can have tangible downstream effects for observational EHR studies. Especially with the increase in electronic visits and telehealth visits since the COVID-19 pandemic (117, 118), there will arise a need to evaluate how well and how often patient health statuses are captured and encoded in this new clinical paradigm.

The care setting in which patients interact with clinicians can also influence whether there is sufficient data for an EHR study. For example, when studying blood biomarkers of cancer, a specialized cancer center may be more likely to collect regular blood tests and cancer stage diagnoses in its database as opposed to EHRs from a general care hospital, making the cancer center EHR system significantly more data-rich. So, while more frequent clinician visits certainly contribute to data quality over time, the type of interaction, whether in a specialized or general care setting, can make a large difference in terms of the data that are available to researchers. It is important to note that patients with severe conditions like cancer may also be more susceptible to fragmentation if they transfer from a general care setting to a specialized one, and this may also influence data richness.

### 4.3. Condition Chronicity and Severity

Chronic diseases are defined as any condition lasting at least 1 year that consistently requires medical attention and/or interferes with daily living (119). Chronic conditions are more likely to be accurately documented in EHR databases than acute conditions for several reasons. If a patient has a chronic condition that interferes with daily living, they may be more likely to visit a clinician and seek medical attention compared to a mild acute illness, such as a cold. This greater amount of captured data can increase study feasibility. Additionally, the longitudinal nature of chronic diseases may make these conditions more robust to data missingness due to EHR fragmentation. Chronic conditions can be present throughout the lifespan, and as such, they can be captured independently at several healthcare systems, even without integration across EHR databases. Of course, this also can depend on the age at which a patient experiences the onset of a chronic disease. For example, if a patient is diagnosed with a chronic condition like lupus at age 10, the diagnosis will likely be captured by EHR systems throughout most of the patient’s life, as opposed to someone who was diagnosed with Alzheimer’s disease at age 80.

Disease severity also varies across chronic and acute conditions, and data captured in the EHR may be affected in several ways because of this. On one hand, there may be less data capture because of the potential for patients to transfer to a specialized care setting if they have a severe chronic condition, as discussed in the previous section. On the other hand, within healthcare systems including general care settings, a severe chronic condition may lead to more routine measurements, such as blood tests for inflammatory markers in autoimmune disease. All in all, it is important to consider the average duration of conditions that are being investigated in an EHR study, as disease chronicity and severity may play a large role in data capture. Investigating the time span of billing codes related to a single condition in an EHR database can also be one way to understand whether proper documentation throughout a condition has occurred prior to beginning a research study.

With conditions varying across so many dimensions that affect the feasibility of an EHR study, it can be useful to map particularly important dimensions out. Figure 5 represents a potential landscape of the interplay of data richness with condition chronicity where various conditions of interest may fall. In this landscape representing a general care setting, a chronic condition like the occurrence of migraines may be more likely to be captured across several healthcare systems and therefore be feasible to study using EHRs, whereas acute conditions that do not require many clinician interactions and are less data rich (e.g., a mild cold) would be more difficult to study.

## 5. ENSURING ROBUST STUDY RESULTS

Once a study group of patients has been constructed and an analysis has been performed, how do researchers make sure their results are robust? In this section, we describe several strategies that researchers can use to support and ensure the robustness of results, divided into internal and external strategies. These strategies can help identify and resolve limitations inherent in EHRs and aid in determining the true generality of observed effects.

### 5.1. Internal Validation

Several robustness checks can be carried out internally in the EHR database from which the original patient study group was constructed. The first of these is sensitivity analyses, which should always be performed. They involve the perturbation of the original study group data followed by repeating analyses to test for any changes in effect that would indicate sensitivity of the conclusions. Data perturbations can include restricting the study group to different age ranges, matching exposed and unexposed patients on comorbidity status, using a different set of controls for comparison, expanding the definition of the disease of interest, matching on the presence of other drug treatments, or regressing on healthcare utilization variables (16, 120). Rerunning analyses after randomly masking a few data points can also help ensure that results are stable, especially for rare conditions. Sensitivity analyses provide researchers the opportunity to take variables into account that they believe may influence study results, either positively or negatively. Ideally, significant results from the initial analysis would remain significant throughout each subsequent sensitivity investigation. If not, the sources of sensitivity must be reported and further explored. Many groups (121, 122) have demonstrated the usefulness of sensitivity analyses in their work, helping define clinical associations between outcomes like comorbidity indices and COVID-19 or myocardial infarction and heart failure that are robust to taking subsets of the patient population and implementing age cutoffs.

Robustness can also be accounted for internally by considering multiple complementary observational study designs. For example, Ramey et al. (16) used both a case-control and cohort study design to look for consistency of an effect of autoimmune diseases on Alzheimer’s disease diagnoses. Using these two study designs to triangulate on autoimmune diseases that were significant, they validated several autoimmune disease subtypes, such as gastrointestinal and endocrine conditions, that may increase Alzheimer’s disease risk. Given both case-control and cohort studies are susceptible to the statistical and data representation biases discussed in Section 2 to different degrees, performing analyses in more than one study design can rule out these biases as the leading cause for any observed effect.

### 5.2. External Validation

External validation, i.e., rerunning an analysis in a different clinical database than the one that was originally used, is a powerful approach to bolster the support for results of observational EHR studies (123, 124). The usage of different EHR billing codes, the provider referral system, and overall documentation of data can vary by hospital and clinic, so external validation is especially useful for determining if study results are due to inherent characteristics of a single healthcare location or if they hold true in other care institutions (125–127). We encourage all EHR researchers to strive for external replication. However, there are many practical and technical barriers to external EHR validation. For example, it is easiest to translate an analysis pipeline to an external database if the database structure is the same as the original. In recent years, many clinical research institutions have adopted the Observational Medical Outcomes Partnership (OMOP) Common Data Model (128, 129), which is an internationally standardized data model that allows for easy transferability of standardized billing codes and data table structures from one institution to the next. OMOP structures have been used at the University of California (UC) to validate results from a single UC institution in a large UC-wide OMOP database that contains integrated data across six UC health centers (23). Similar validation studies have been performed linking OMOP data across healthcare centers at UC San Francisco and Stanford (16), both of which use OMOP, to calculate Alzheimer’s risk as well as a mapping across several databases to an OMOP structure for the contextualization of COVID-19 adverse events (130).

In contrast to OMOP, many institutions rely on internal proprietary research data warehouse structures instead of a widely used data model. While these can make it difficult to directly transfer an analysis between locations, in these cases, researchers can maintain a list of diagnosis, procedural, and other medical billing codes used in their original analysis and pick an external database that also maintains the same billing code ontologies. In this case, while the data table structures of a database may be different, the underlying billing codes should remain consistent. The World Health Organization–based ICD terminology is widely used throughout many data warehouses, along with others such as Systematized Nomenclature of Medicine terminology (131, 132). Ontologies that consolidate billing codes into larger clinically meaningful categories such as phecodes (84) are also useful tools for transferring diagnoses between databases and can circumvent issues of differential code usage by healthcare location. All of these ontologies may be present in other academic institutions’ data warehouses or in national clinical biobanks such as the All of Us database (https://allofus.nih.gov).

## 6. CONCLUSIONS AND SUGGESTIONS FOR THE FUTURE

Here we have discussed potential biases in observational EHR studies due to provider, patient, and data structure characteristics. We bring up several ways to identify and address these challenges, including bias quantification measures, statistical matching techniques, and careful cohort and database selection practices. We also draw attention to several dimensions along which conditions and diseases in the EHR can vary, making them more or less feasible to study. To our knowledge, this aspect of EHR study feasibility has not been thoroughly explored, and we are eager for future work on our hypotheses about the disease dimensions that enable EHR study. Study reproducibility is also a major problem in the EHR field. We put forth internal and external validation strategies that researchers can use to increase the robustness of their results, and we encourage researchers to seek external validation as a standard for publishing EHR research.

EHR studies provide a valuable real-world data source to clinical medicine and the scientific research community. With larger EHR databases and better accounting for and reporting of biases in EHR data, we anticipate that they will have even greater impacts in the coming years. As EHRs are an essential resource for biomedical research, there is great potential for their further integration with other data modalities such as genomics, dental and vision records, and data from personal health monitors to provide a holistic picture of a patient’s health trajectory, which will ultimately lead to better patient outcomes.

## Figures and Tables

**Figure 1:**
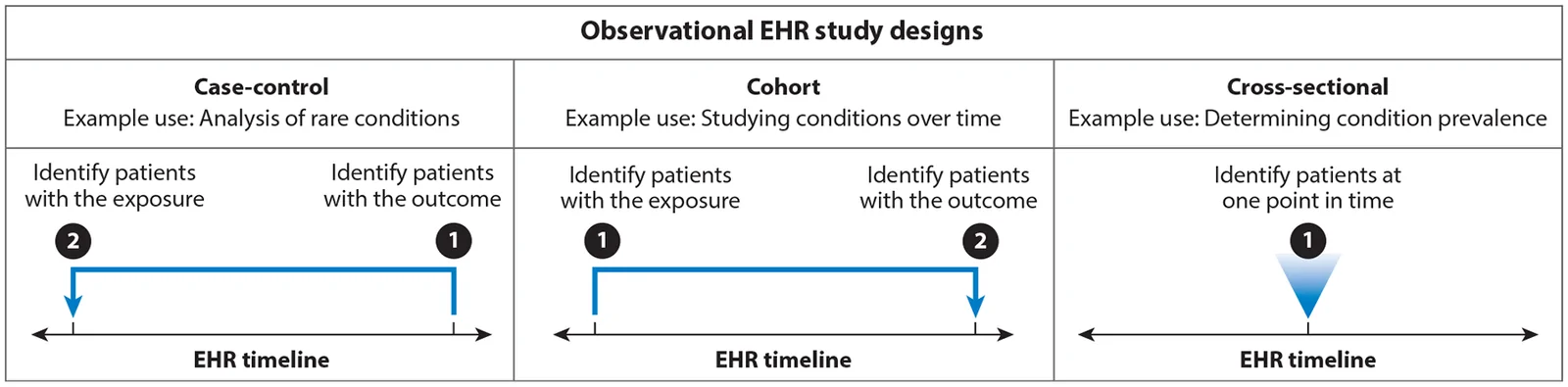
Overview of observational EHR study designs. Three study designs are common in EHR studies: case-control, cohort, and cross-sectional studies. Each has its own advantages based on the nature of inference a researcher is conducting. In each panel, ① represents the first step conducted in study group creation, followed by step ②. (Note that there is no step ② for cross-sectional studies because these involve a single time point in the EHR.) Abbreviation: EHR, electronic health record.

**Figure 2:**
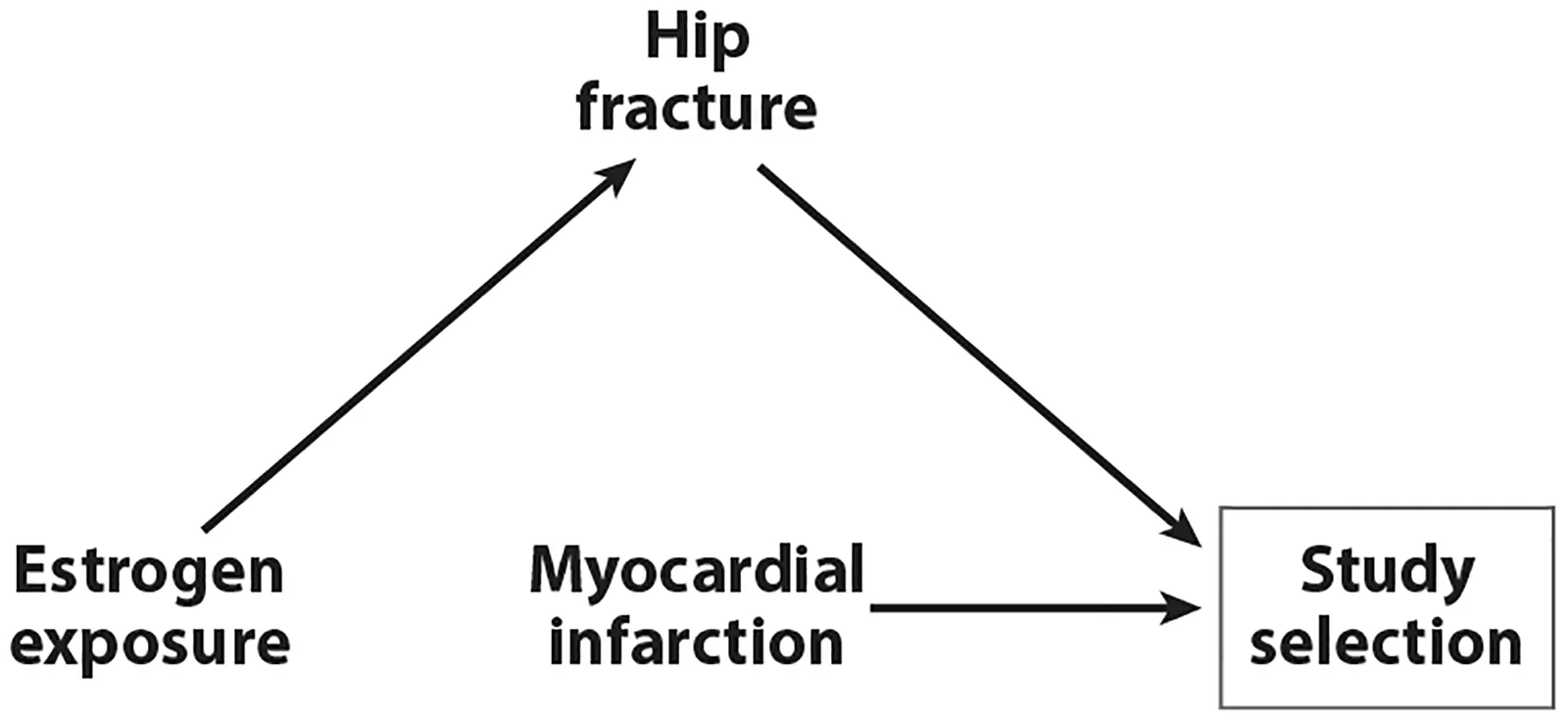
Example DAG to identify variables influencing study selection. This DAG represents how hip fractures affect selection of patients in a case-control study of the effects of estrogen exposure on myocardial infarction (28). Abbreviation: DAG, directed acyclic graph.

**Figure 3:**
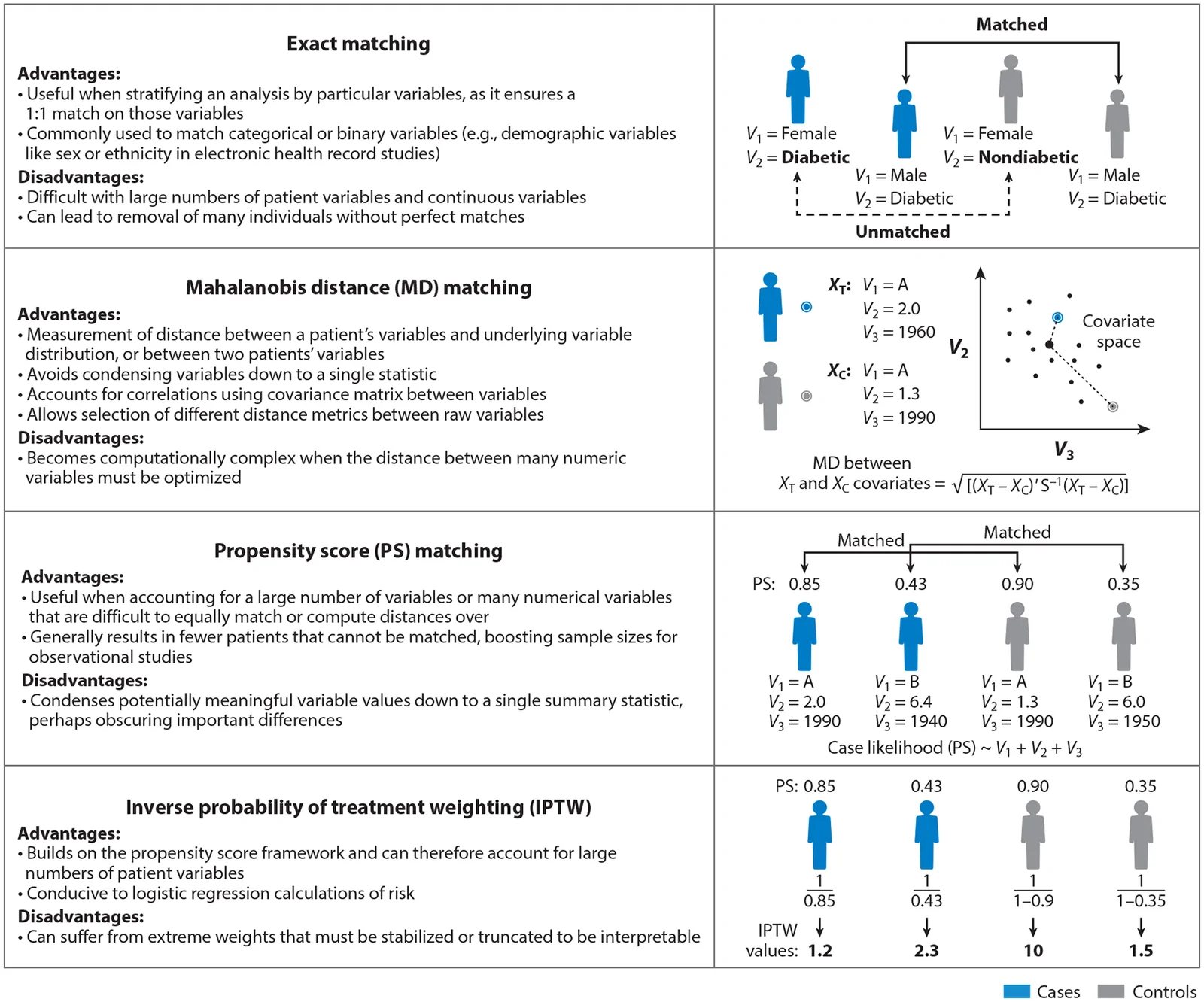
Study group matching and weighting methods. Diagrams on the right represent a graphical overview of how each method is conducted. Each method has advantages and disadvantages related to how many covariates a researcher is interested in accounting for, computational efficiency limits, and potential downstream applications of the matched study groups. Generally, methods at the top can obtain closer matches or retain meaningful variable differences at the expense of being more computationally complex, while methods at the bottom can account for large numbers of variables, result in fewer removed patients, and are more computationally efficient. Methods can also often be used in combination for different variables. Variables: *S*, covariance matrix; *V*_i_, patient variables; *X*_C_, control patient variable vector; *X*_T_, treated/case patient variable vector.

**Figure 4:**
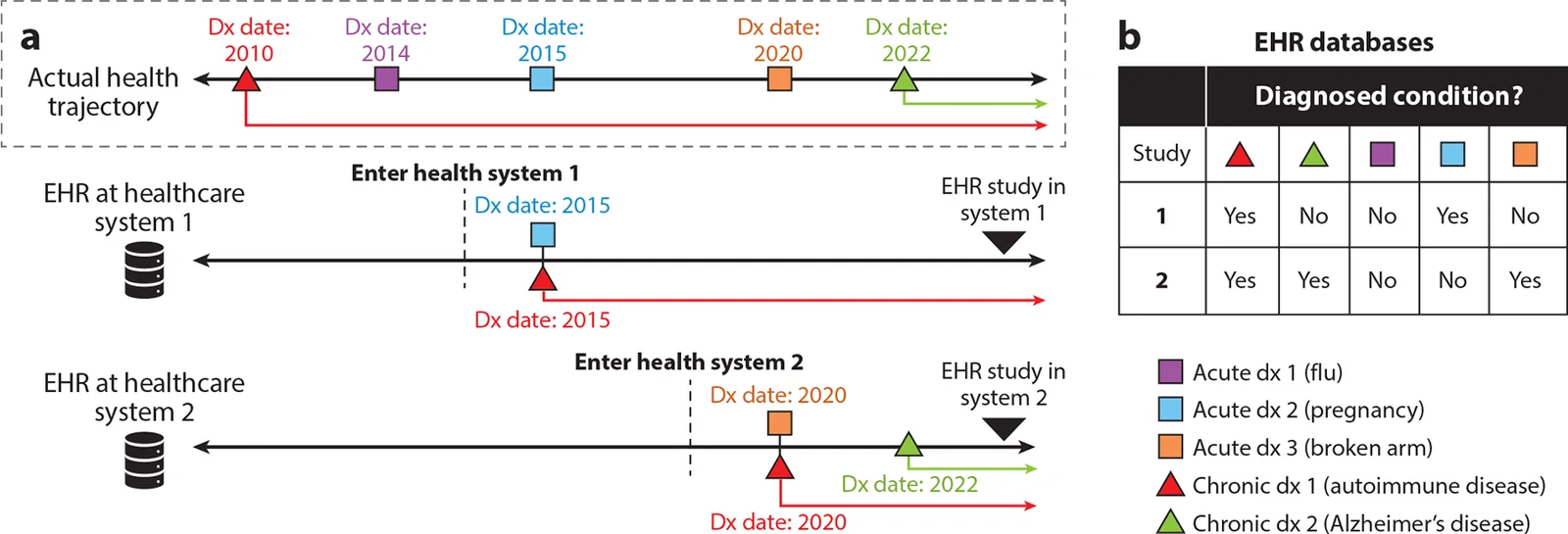
Schematic of challenges posed by data fragmentation across EHR systems. (*a*) Example of the complete health trajectory of a patient versus what is captured in two EHR systems used by the patient. Both chronic (*triangles*) and acute (*squares*) diagnoses can be missed due to data fragmentation across EHRs. (*b*) Missing data due to fragmentation of records across different healthcare systems, where some but not all of the data are represented for a patient, can influence downstream EHR analyses. For example, chronic diseases may be more likely to be accurately phenotyped given their presence across much of the lifespan; however, the documentation of when the disease appeared may be less accurate. Abbreviations: dx, diagnosis; EHR, electronic health record.

**Figure 5:**
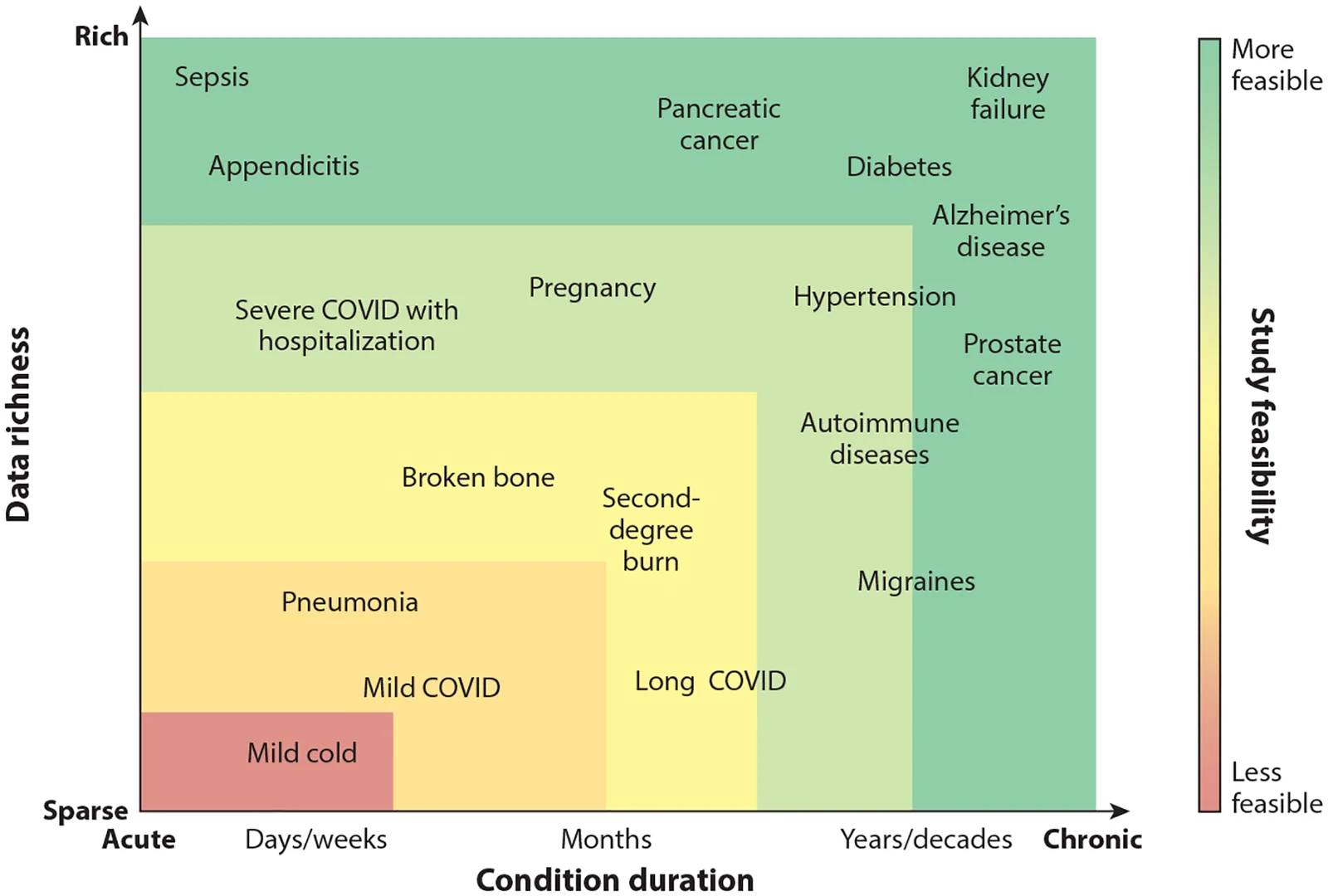
Retrospective EHR study feasibility varies across conditions. Data richness and condition duration are two major factors that determine how often data are collected on patients and therefore whether there are enough accurate data to conduct a study. Severe conditions (e.g., sepsis) often have greater data richness due to the intensity of care required and therefore greater documentation produced. Severe conditions thus often exhibit high feasibility for EHR studies. Alternatively, a mild cold would be less likely to be documented with accuracy across several healthcare systems. Abbreviation: EHR, electronic health record.
